# Visual Analysis of Dynamics Behaviour of an Iterative Method Depending on Selected Parameters and Modifications

**DOI:** 10.3390/e22070734

**Published:** 2020-07-02

**Authors:** Ireneusz Gościniak, Krzysztof Gdawiec

**Affiliations:** Institute of Computer Science, University of Silesia, Bȩdzińska 39, 41–200 Sosnowiec, Poland; krzysztof.gdawiec@us.edu.pl

**Keywords:** root finding, dynamics, iterations, visualisation

## Abstract

There is a huge group of algorithms described in the literature that iteratively find solutions of a given equation. Most of them require tuning. The article presents root-finding algorithms that are based on the Newton–Raphson method which iteratively finds the solutions, and require tuning. The modification of the algorithm implements the best position of particle similarly to the particle swarm optimisation algorithms. The proposed approach allows visualising the impact of the algorithm’s elements on the complex behaviour of the algorithm. Moreover, instead of the standard Picard iteration, various feedback iteration processes are used in this research. Presented examples and the conducted discussion on the algorithm’s operation allow to understand the influence of the proposed modifications on the algorithm’s behaviour. Understanding the impact of the proposed modification on the algorithm’s operation can be helpful in using it in other algorithms. The obtained images also have potential artistic applications.

## 1. Introduction

Most of the algorithms determining the local extremes and especially the minimum of the function F are based on determining the value of the function or its gradient. In the case of such an algorithm, the new position of the particle is calculated basing on its previous position and the gradient which determine the direction of the movement [1]—an example of this approach is the gradient descent method:

(1) $$z_{i}^{\prime }=z_{i}-\gamma \nabla F\left(z_{i}\right),$$

where: −∇F—negative gradient, γ—step size, ${z^{\prime }}_{i}$—the current position of the *i*th particle in a *D* dimensional environment, $z_{i}$—the previous position of the *i*th particle. There are many algorithms implementing modifications of this method, and its operation is well described in the literature.

Evolutionary algorithms are another group of algorithms used to solve optimisation problems, including minimisation. As shown in [2], the analysis of particle behaviour in the evolutionary algorithms is a very complex problem. In practice, many algorithms similar to the evolutionary algorithms are used [3]. Particularly noteworthy is the group of particle swarm optimisation algorithms (PSO) [4]. The complex nature of the interaction between particles does not allow us to determine their impact on the operation of the algorithm in a simple way. The behaviour of particles in the PSO algorithm can be described by the following equation:

(2) $$z_{i}^{\prime }=z_{i}+v_{i}^{\prime },$$

where: $z_{i}^{\prime }$—the current position of the *i*th particle in a *D* dimensional environment, $z_{i}$—the previous position of the *i*th particle, $v_{i}^{\prime }$—the current velocity of the *i*th particle in a *D* dimensional environment that is given by the following formula:

(3) $$v_{i}^{\prime }=\omega v_{i}+\eta_{1}r_{1}\left(z_{pbest\;i}-z_{i}\right)+\eta_{2}r_{2}\left(z_{gbest}-z_{i}\right),$$

where: $v_{i}$—the previous velocity of the *i*th particle, ω—inertia weight (ω∈[0,1]), $\eta_{1}$, $\eta_{2}$—acceleration constants ($\eta_{1},\eta_{2}\in \left(0,1\right]$), $r_{1}$, $r_{2}$—random numbers generated uniformly in the [0,1] interval, $z_{pbest\;i}$—the best position of the *i*th particle, $z_{gbest}$—the global best position of the particles. The best position of the particle and the best global position are calculated in each iteration. The position of the particle and the best global position have a significant impact on the behaviour of not only the particle, but the entire swarm, directing them towards the best solution. The particle behaviour is determined by three parameters: inertia weight (ω) and two acceleration constants ($\eta_{1},\eta_{2}$). The PSO algorithm belongs to the group of stochastic algorithms for which the convergence can be proved [5]. The ω parameter informs about the influence of the particle velocity at the moment t−1 on its velocity at the moment *t*. On the other hand, the η parameter affects the changes in the particle acceleration at the time *t*. For ω<1 the particles slow down to reach the velocity of zero. For the value of ω close to 0.7 and the value of $\eta_{1}$ and $\eta_{2}$ close to 1.5 the PSO algorithm has good convergence. The cooperation of particles releases an adaptive mechanism that drives the particle and determines its dynamics. The selection of the proper values of inertia weight and acceleration constants is a very important problem for the algorithm [6]—the algorithm tuning is intuitive and based on developer’s experience.

Another important problem that arises in practice is finding the solutions of a system of non-linear equations. Due to many difficulties in solving such systems by using analytical methods many numerical methods were developed. The most famous method of this type is the Newton’s method [7]. In recent years methods with different approaches were proposed. In [8] Goh et al. inspired by the simplex algorithm, proposed a partial Newton’s method. Despite this method is based on Newton’s method, it has slower convergence. Another method based on Newton’s method was proposed in [9]. In this method Saheya et al. revised the Jacobian matrix by a rank one matrix each iteration. Sharma et al. in [10] introduced a three-step iterative scheme that is based on Newton–Traub’s method. A method that is based on a Jarratt-like method was proposed by Abbasbandy et al. in [11]. This method uses only one LU factorisation which preserves and reduces computational complexities. An acceleration technique that can be used in many iterative methods was proposed in [12]. Moreover, the authors introduced a new family of two-step third order methods for solving systems of non-linear equations. Recently, Alqahtani et el. proposed a three-step family of iterative methods [13]. This family is a generalisation of the fourth-order King’s family to the multidimensional case. Awwal et al. in [14] proposed a two-step iterative algorithm that is based on projection technique and solves system of monotone non-linear equations with convex constraints.

In addition to the methods based on classical root finding methods (Newton, Traub, Jarratt, etc.), in the literature we can find approaches based on the PSO algorithm and other metaheuristic algorithms. In [15] Wang et al. proposed a modification of the PSO algorithm in which the search dynamics is controlled by a traditional controller such as the PID (Proportional–Integral–Derivative) controller. A combination of Nelder–Mead simplex method and PSO algorithm was proposed in [16]. To overcome problems of being trapped in local minima and slow convergence, Jaberipour et al. proposed a new way of updating particle’s position [17]. Another modification of the PSO algorithm for solving systems of non-linear equations was proposed by Li et al. in [18]. The authors proposed: (1) a new way of inertia weight selection, (2) dynamics conditions of stopping iterating, (3) calculation of the standardised number of restarting times based on reliability theory. Ibrahim and Tawhid in [19] introduced a hybridisation of cuckoo search and PSO for solving system of non-linear equations, and in [20] a hybridisation of differential evolution and the monarch butterfly optimisation. Recently, Liao et al. in [21] introduced a decomposition re-initialisation-based differential evolution to locate multiple roots of non-linear equations system. A study on different metaheuristic methods, namely PSO, firefly algorithm and cuckoo search algorithm, in the solving of system of non-linear equation task was conducted in [22]. The study showed that the PSO algorithm obtains better results than the other two algorithms.

Dynamic analysis is a separate field of research using numerous investigation methods [23]. Among them, there is a graphical visualisation of dynamics [24]. In the analysis of the complex polynomial root finding process the polynomiography is a very popular method of visualising the dynamics [25,26,27]. In this method the number of iterations required to obtain the solution is visualised. The generated images show the dynamics of the root-finding process using some colour maps. Today, dynamics study of root-finding methods with polynomiography is an essential part of modern analysis of the quality of these methods [28]. It turns out that this form of dynamics study is a powerful tool in selecting optimal parameters that appear in the iterative methods for solving non-linear equations and to compare these methods [29,30]. The visualisation methods proposed in the work will also be called polynomiography because of their similarity to the primary polynomiography, despite the fact that we use any functions, not just polynomials.

This article proposes modifications of a root finding algorithm, that is based on the Newton’s method, by using the inertia weight and the acceleration constant as well as the best position of the particle and the implementation of various iteration methods. The conducted research serves to visualise the impact of the proposed algorithm modifications on the behaviour of the particle. They allow conducting not only the discussion on the behaviour of a particle, but also to present images of aesthetic character and artistic meaning [31,32].

Nammanee et al. [33] introduced a strong convergence theorem for the modified Noor iterations in the framework of uniformly smooth Banach spaces. Their results allow to suppose that the proposed modifications using the best particle position will be an effective way to increase the effectiveness of algorithm’s operation.

The rest of the paper is organised as follows. Section 2 introduces root finding algorithm that is based on the Newton–Raphson method. This method is used to illustrate the tuning influence on its behaviour. Next, Section 3 introduces iteration processes known in literature. Moreover, basing on these iterations we propose iterations that use the best position of the particle. Section 4 presents algorithms for creating polynomiographs. Then, in Section 5 a discussion on the research results illustrated by the obtained polynomiographs is made. Finally, Section 6 gives short concluding remarks.

## 2. The Algorithm

Numerous methods can be used to solve a system of *D* non-linear equations with *D* variables. One of the best known and intensively studied methods is the Newton–Raphson method [7]. Let $f_{1},f_{2},\ldots ,f_{D}:R^{D}\to R$ and let

(4) $$F\left(z^{1},z^{2},\ldots ,z^{D}\right)=\left[\begin{array}{c} f_{1}\left(z^{1},z^{2},\ldots ,z^{D}\right)\\ f_{2}\left(z^{1},z^{2},\ldots ,z^{D}\right)\\ \vdots \\ f_{D}\left(z^{1},z^{2},\ldots ,z^{D}\right) \end{array}\right]=\left[\begin{array}{c} 0\\ 0\\ \vdots \\ 0 \end{array}\right]=0.$$

Let $F:R^{D}\to R^{D}$ be a continuous function which has continuous first partial derivatives. Now, to solve the equation F(z)=0, where $z=[z^{1},z^{2},\ldots ,z^{D}]$, by using the Newton–Raphson method, a starting point is selected $z_{0}=\left[z_{0}^{1},z_{0}^{2},\ldots ,z_{0}^{D}\right]$ and then the iterative formula is used as follows:

(5) $$z_{n+1}=z_{n}-J^{-1}\left(z_{n}\right)F\left(z_{n}\right)\;n=0,1,2,\ldots ,$$

where:

(6) $$J\left(z\right)=\left[\begin{array}{cccc} \frac{\partial f_{1}}{\partial z_{1}}\left(z\right) & \frac{\partial f_{1}}{\partial z_{2}}\left(z\right) & \ldots & \frac{\partial f_{1}}{\partial z_{D}}\left(z\right)\\ \frac{\partial f_{2}}{\partial z_{1}}\left(z\right) & \frac{\partial f_{2}}{\partial z_{2}}\left(z\right) & \ldots & \frac{\partial f_{2}}{\partial z_{D}}\left(z\right)\\ \vdots & \vdots & \vdots & \vdots \\ \frac{\partial f_{D}}{\partial z_{1}}\left(z\right) & \frac{\partial f_{D}}{\partial z_{2}}\left(z\right) & \ldots & \frac{\partial f_{D}}{\partial z_{D}}\left(z\right) \end{array}\right]$$

is the Jacobian matrix of F and $J^{-1}$ is its inverse.

By taking $N\left(z\right)=-J^{-1}\left(z\right)F\left(z\right)$ the Newton–Raphson method can be described as follows:

(7) $$z_{n+1}=z_{n}+N\left(z_{n}\right),\;n=0,1,2,\ldots .$$

To solve (4), the following algorithm can be used:

(8) $$z_{n+1}=z_{n}+v_{n+1},$$

where: $z_{0}\in R^{D}$ is a starting position, $v_{0}=\left[0,0,\ldots ,0\right]$ is a starting velocity, $v_{n+1}$ is the current velocity of particle ($v_{n+1}=\left[v_{n+1}^{1},v_{n+1}^{2},\ldots ,v_{n+1}^{D}\right]$), $z_{n}$ is the previous position of particle ($z_{n}=\left[z_{n}^{1},z_{n}^{2},\ldots ,z_{n}^{D}\right]$). The algorithm sums the position of the particle $z_{n}$ with its current velocity $v_{n+1}$. The current velocity of the particle is determined by the inertia weight and the acceleration constants:

(9) $$v_{n+1}=\omega v_{n}+\eta N\left(z_{n}\right),$$

where: $v_{n}$—the previous velocity of particle, ω∈[0,1)—inertia weight, η∈(0,1]—acceleration constant. Let us notice that (8) reduces to the classical Newton’s method [25] if ω=0 and η=1, and to the relaxed Newton’s method [34] if ω=0 and η≠1. Moreover, we can observe that the proposed method joins the PSO algorithm and the Newton’s method. The acceleration term in the PSO algorithm is responsible for pointing the particle in the direction of the best solution. In our method as the acceleration term we use the direction used in the Newton’s method, which is responsible for moving the current point into direction of the solution. Thus, in each iteration we are moving towards the solution and eventually we will stop in the solution, because for ω<1 the particle slows down and $N(z_{n})=0$, when we reach the solution.

The implementation of inertia weight (ω) and the acceleration constant (η) allows controlling particle dynamics in a wider range [4]. The selection of the value of these parameters is not deterministic. It depends on the problem being solved and the selected iteration method. These values are selected by tuning—it is a kind of art. The relationships between the ω and η parameters show a complex nature and the change of these parameters influences particle’s dynamics, which can be visualised using algorithms described in Section 4.

A very important feature in numerical methods for solving systems of equations is the order of convergence of the method. For instance, the order of convergence of Newton’s method is 2 [25]. In the case of the methods that are based on Newton’s, Traub, Jarratt, etc. methods the order of convergence is proved (see for instance [8,9,10,11,12,13,14]), but in case of the methods that are based on metaheuristic algorithms, like the method presented in this paper, the order of convergence is not derived (see for instance [15,16,17,18,19,20,21,22]). This is due the fact that this order greatly varies for different values of the parameters used in the metaheuristic algorithms. We will show this on a very simple example. In this example to approximate the order of convergence we will use the computational order convergence introduced in [35], i.e.,

(10) $$p\approx \frac{\ln\left(∥z_{n+1}-z_{n}∥/∥z_{n}-z_{n-1}∥\right)}{\ln\left(∥z_{n}-z_{n-1}∥/∥z_{n-1}-z_{n-2}∥\right)}.$$

Let us consider $f_{1}\left(x,y\right)=x^{3}-3xy^{2}-1$, $f_{2}\left(x,y\right)=3x^{2}y-y^{3}$. We will solve system (4) by using (8) with various values of ω and η in (9) and three different starting points $z_{0}$. The obtained results are presented in Table 1. For ω=0 and η=1 (classic Newton’s method) we see that the method obtains second order of convergence, which is in accordance with the result proved in the literature. When we take a non-zero inertia, then we see that for a fixed starting point and varying ω and η we obtain very different values of the order. The same situation happens when we fix ω and η and change the starting points.

Another problem studied in the context of solving systems of non-linear equation with iterative methods is the problem of strange (extraneous) fixed points, i.e., fixed points which are found by the method and are not the roots of the considered system [36]. Let us assume that (8) has converged to a solution. In such case the $v_{n}$ in (9) is equal to 0. Therefore, method (8) behaves like relaxed Newton’s method. As a result that relaxed Newton’s method has no strange fixed point [25], the proposed method also has no strange fixed points.

## 3. Iteration Processes

Picard’s iteration [37] is widely used in many iterative algorithms. This iteration can be described as follows:

(11) $$z_{n+1}=T\left(z_{n}\right).$$

Notice that (8) uses the Picard iteration, where $T:R^{D}\to R^{D}$ takes the form

(12) $$T\left(z_{n}\right)=z_{n}+v_{n+1}.$$

In the literature many other iteration processes can be found. Three most basic and commonly implemented algorithms of the approximate finding of fixed points of mappings are as follows:

1. The Mann iteration [38]:
  (13) $$z_{n+1}=\left(1-\alpha_{n}\right)z_{n}+\alpha_{n}T\left(z_{n}\right),\;n=0,1,2,\ldots ,$$
  where: $\alpha_{n}\in \left(0,1\right]$ for all n∈N, and the Mann iteration for $\alpha_{n}=1$ reduces to the Picard iteration.
2. The Ishikawa iteration [39]:
  (14) $$\begin{array}{cc} z_{n+1} & =\left(1-\alpha_{n}\right)z_{n}+\alpha_{n}T\left(u_{n}\right),\\ u_{n} & =\left(1-\beta_{n}\right)z_{n}+\beta_{n}T\left(z_{n}\right),\;n=0,1,2,\ldots , \end{array}$$
  where: $\alpha_{n}\in \left(0,1\right]$ and $\beta_{n}\in \left[0,1\right]$ for all n∈N, the Ishikawa iteration reduces to the Mann iteration when $\beta_{n}=0$, and to the Picard iteration when $\alpha_{n}=1$ and $\beta_{n}=0$.
3. The Agarwal iteration [40] (*S*-iteration):
  (15) $$\begin{array}{cc} z_{n+1} & =\left(1-\alpha_{n}\right)T\left(z_{n}\right)+\alpha_{n}T\left(u_{n}\right),\\ u_{n} & =\left(1-\beta_{n}\right)z_{n}+\beta_{n}T\left(z_{n}\right),\;n=0,1,2,\ldots , \end{array}$$
  where $\alpha_{n}\in \left[0,1\right]$ and $\beta_{n}\in \left[0,1\right]$ for all n∈N, let us notice that the *S*-iteration reduces to the Picard iteration when $\alpha_{n}=0$, or $\alpha_{n}=1$ and $\beta_{n}=0$.

A survey of various iteration processes and their dependencies can be found in [41].

An additional particle (a sample in the solution space) $u_{n}$ (acting as a reference point) is applied in the Ishikawa and Agarwal iterations. In this case, each of these iterations performs two steps. The reference point $u_{n}$ is set in the first step, and next a new particle position $z_{n+1}$ is computed using the reference point. It allows better control of the particle’s motion. In the paper a modification of the above-discussed iterations is proposed by applying the best position of the reference point and the best position of the particle—similar to the PSO algorithm.

The positions of the reference point $u_{n}$ and the particle $z_{n}$ are evaluated and compared to $u_{best}$ and $z_{best}$. In solutions space, the lower value of ∥F(q)∥ determines the better position of the reference point or the particle (q). The next $u_{best}$ and $z_{best}$ are updated if they are worse than $u_{n}$ and $z_{n}$. The best position of the reference point $u_{best}$ or position of the reference point $u_{n}$ and the best position of the particle $z_{best}$ or the position of the particle $z_{n}$ (depending on the result of the evaluation) are used to determine the new position of the particle $z_{n+1}$.

As a result of this modification, the next position of the particle $z_{n+1}$ in the Equations (13)–(15) is determined by the following formulas:

1. The modified Mann iteration
  (16) $$z_{n+1}=\left(1-\alpha_{n}\right)z_{\left\{n|best\right\}}+\alpha_{n}T\left(z_{n}\right),$$
2. The modified Ishikawa iteration
  (17) $$z_{n+1}=\left(1-\alpha_{n}\right)z_{\left\{n|best\right\}}+\alpha_{n}T\left(u_{\left\{n|best\right\}}\right),$$
3. The modified Agarwal iteration
  (18) $$z_{n+1}=\left(1-\alpha_{n}\right)T\left(z_{\left\{n|best\right\}}\right)+\alpha_{n}T\left(u_{\left\{n|best\right\}}\right).$$

All the presented iterations used only one mapping, but in the fixed point theory exist iterations that use several mappings and are applied to find common fixed points of the mappings. Examples of this type of iterations are the following:

1. The Das–Debata iteration [42]:
  (19) $$\begin{array}{cc} z_{n+1} & =\left(1-\alpha_{n}\right)z_{n}+\alpha_{n}T_{2}\left(u_{n}\right),\\ u_{n} & =\left(1-\beta_{n}\right)z_{n}+\beta_{n}T_{1}\left(z_{n}\right),\;n=0,1,2,\ldots , \end{array}$$
  where $\alpha_{n}\in \left(0,1\right]$ and $\beta_{n}\in \left[0,1\right]$ for all n∈N, the Das–Debata iteration for $T_{1}=T_{2}$ reduces to the Ishikawa iteration.
2. The Khan–Cho–Abbas iteration [43]:
  (20) $$\begin{array}{cc} z_{n+1} & =\left(1-\alpha_{n}\right)T_{1}\left(z_{n}\right)+\alpha_{n}T_{2}\left(u_{n}\right),\\ u_{n} & =\left(1-\beta_{n}\right)z_{n}+\beta_{n}T_{1}\left(z_{n}\right),\;n=0,1,2,\ldots , \end{array}$$
  where $\alpha_{n}\in \left(0,1\right]$ and $\beta_{n}\in \left[0,1\right]$ for all n∈N, let us notice that the Khan–Cho–Abbas iteration reduces to the Agarwal iteration when $T_{1}=T_{2}$.
3. The generalised Agarwal’s iteration [43]:
  (21) $$\begin{array}{cc} z_{n+1} & =\left(1-\alpha_{n}\right)T_{3}\left(z_{n}\right)+\alpha_{n}T_{2}\left(u_{n}\right),\\ u_{n} & =\left(1-\beta_{n}\right)z_{n}+\beta_{n}T_{1}\left(z_{n}\right),\;n=0,1,2,\ldots , \end{array}$$
  where $\alpha_{n}\in \left(0,1\right]$ and $\beta_{n}\in \left[0,1\right]$ for all n∈N, moreover the generalised Agarwal iteration reduces to the Khan–Cho–Abbas iteration when $T_{1}=T_{3}$ and to the Agarwal iteration when $T_{1}=T_{2}=T_{3}$.

Iterations described by Equations (19)–(21) can be also modified by introducing the best position of the reference point and the best position of the particle. As a result of this modification, equations describing $z_{n+1}$ take the following form:

1. The modified Das–Debata iteration
  (22) $$z_{n+1}=\left(1-\alpha_{n}\right)z_{\left\{n|best\right\}}+\alpha_{n}T_{2}\left(u_{\left\{n|best\right\}}\right),$$
2. The modified Khan–Cho–Abbas iteration
  (23) $$z_{n+1}=\left(1-\alpha_{n}\right)T_{1}\left(z_{\left\{n|best\right\}}\right)+\alpha_{n}T_{2}\left(u_{\left\{n|best\right\}}\right),$$
3. The modified generalised Agarwal iteration
  (24) $$z_{n+1}=\left(1-\alpha_{n}\right)T_{3}\left(z_{\left\{n|best\right\}}\right)+\alpha_{n}T_{2}\left(u_{\left\{n|best\right\}}\right).$$

For all iterations $z_{n}$ is used for the base type of iteration (Algorithm 1), $u_{best}$ is used for the proposed algorithm with the best reference point (Algorithm 2), $z_{best}$ is used for the proposed algorithm with the best particle position (Algorithm 3) and both $u_{best}$ and $z_{best}$ are used for the proposed Algorithm 4. The variety of different iteration processes is implemented in our algorithms. In the iterations we use (12) as the mapping with different values of ω and η parameters.

**Algorithm 1:** Visualisation of the dynamics—the base Algorithm (I)

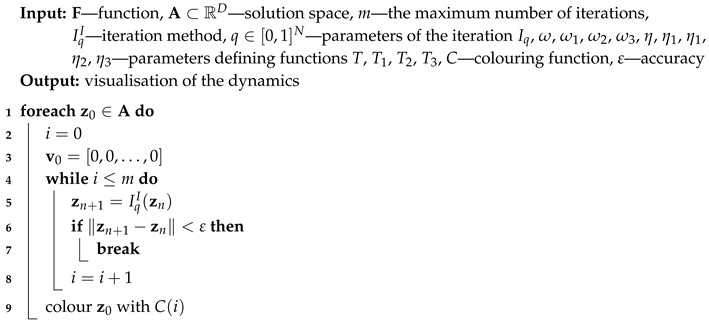



**Algorithm 2:** Visualisation of the dynamics with the best reference point (II)

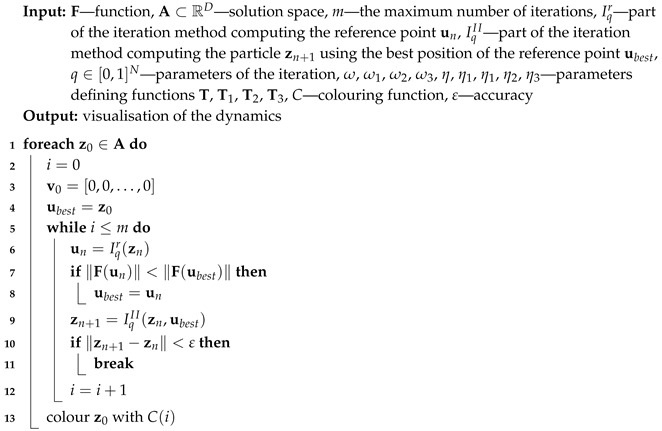



**Algorithm 3:** Visualisation of the dynamics with the best particle (III)

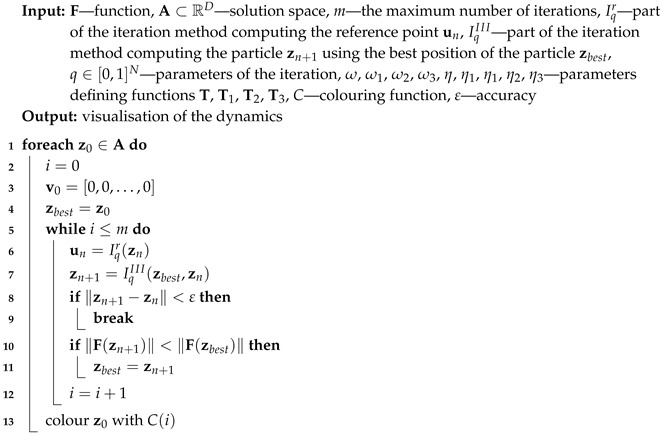



**Algorithm 4:** Visualisation of the dynamics with both the best reference point and particle (IV)

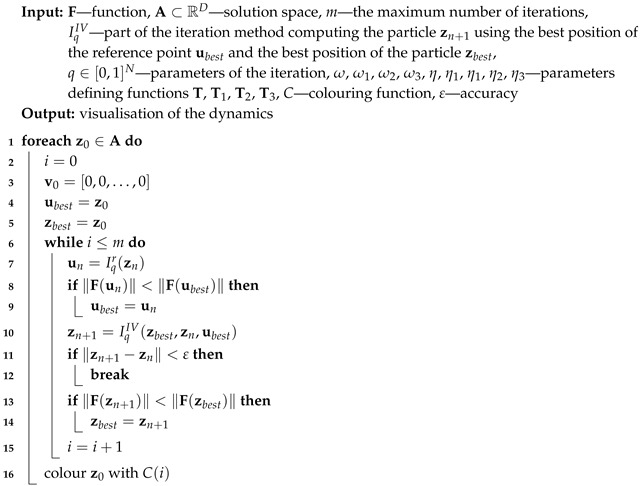



## 4. Visualisation of the Dynamics

A very similar method to the polynomiography [44] is used to visualise the dynamics of the proposed algorithms. The iteration methods presented in Section 3 are implemented in the algorithms and proper values of parameters are selected. ω, η for a single mapping T or $\omega_{1},\omega_{2},\omega_{3}$ and $\eta_{1},\eta_{2},\eta_{3}$ for $T_{1},T_{2},T_{3}$ (depending on the chosen iteration). The maximum number of iterations *m* which algorithm should make, accuracy of the computations ε and a colouring function $C:N\to {\left\{0,1,\ldots ,255\right\}}^{3}$ are set. Then, each $z_{0}$ in the solution space A is used in the algorithm. The iterations of the algorithm proceed till the convergence criterion:

(25) $$∥z_{n+1}-z_{n}∥<\varepsilon$$

or the maximum number of iterations is reached. A colour corresponding to the performed number of iterations is assigned to $z_{0}$ using colouring function *C*. Iterations described in Section 3 and their modifications using the best particle position and the best reference point position allow proposing five algorithms. Algorithm 1 (denoted in the text by *I*) is the base algorithm, without modification. Algorithm 2 (denoted in the article by II) performs the selection of the best position of the reference point. Algorithm 3 (denoted by III) performs the selection of the best particle. Algorithm 4 (denoted by IV) combines the modifications introduced in Algorithms 2 and 3. Algorithm 5 is a modification of Algorithm 3 because Mann iteration does not use a reference point and it is denoted by IIIM.
**Algorithm 5:** Visualisation of the dynamics with the best particle for the Mann iteration (IIIM)
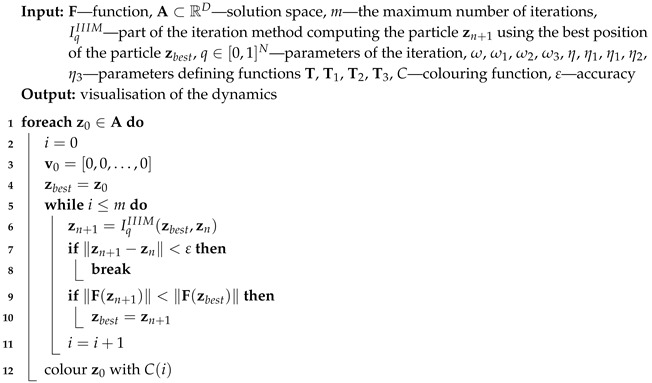


The iteration method is denoted by $I_{q}^{a}$ for a given algorithm, where *q* is a vector of parameters of the iteration and *a* is one of the algorithms {I|II|III|IIIM|IV} or *r*—it is the part of iteration determining position of the reference point.

The domain (solution space) A is defined in a *D*-dimensional space, thus the algorithms return polynomiographs in this space. For D=2 a single image is obtained or for D>2 a two-dimensional cross-section of A can be selected for visualisation.

## 5. Discussion on the Research Results

In this section we present and discuss the obtained results of visualising the dynamics of the method introduced in Section 2 together with the various iteration methods presented in Section 3 using algorithms described in Section 4. Let C be the field of complex numbers with a complex number c=x+iy where $i=\sqrt{-1}$ and x,y∈R. In the experiments we want to solve the following non-linear equation

(26) p(c)=0

where $p\left(c\right)=c^{3}-1$.

This equation can be written in the following form:

(27) $$0=c^{3}-1={\left(x+iy\right)}^{3}-1=x^{3}-3xy^{2}-1+\left(3x^{2}y-y^{3}\right)i.$$

Now, Equation (27) can be transformed into a system of two equations with two variables, i.e.,

(28) $$F\left(x,y\right)=\left[\begin{array}{c} f_{1}\left(x,y\right)\\ f_{2}\left(x,y\right) \end{array}\right]=\left[\begin{array}{c} 0\\ 0 \end{array}\right]=0,$$

where $f_{1}\left(x,y\right)=x^{3}-3xy^{2}-1$, $f_{2}\left(x,y\right)=3x^{2}y-y^{3}$. The set of solutions of this system is the following: [1,0], [−0.5,−0.866025], [−0.5,0.866025] and $A={\left[-2.0,2.0\right]}^{2}$ (${\left[-0.5,0.5\right]}^{2}$ is used for magnification of the centre parts of polynomiographs).

Moreover, several additional test functions are used:

(29) $$\begin{array}{cc} 0 & =c^{4}-10c^{2}+9={\left(x+iy\right)}^{4}-10{\left(x+iy\right)}^{2}+9\\ \; & =x^{4}-6x^{2}y^{2}+y^{4}-10x^{2}+10y^{2}+9+\left(4x^{3}y-4xy^{3}-20xy\right)i, \end{array}$$

where: $f_{1}\left(x,y\right)=x^{4}-6x^{2}y^{2}+y^{4}-10x^{2}+10y^{2}+9$, $f_{2}\left(x,y\right)=4x^{3}y-4xy^{3}-20xy$ and the set of solutions of this system is the following: [−3.0,0.0], [−1.0,0.0], [1.0,0.0], [3.0,0.0] and A=[−4.0,4.0]×[−2.0,2.0];

(30) $$\begin{array}{cc} 0 & =c^{5}-c={\left(x+iy\right)}^{5}-\left(x+iy\right)\\ \; & =x^{5}-10x^{3}y^{2}+5xy^{4}-x+\left(5x^{4}y-10x^{2}y^{3}+y^{5}-y\right)i, \end{array}$$

where: $f_{1}\left(x,y\right)=x^{5}-10x^{3}y^{2}+5xy^{4}-x$, $f_{2}\left(x,y\right)=5x^{4}y-10x^{2}y^{3}+y^{5}-y$ and the set of solutions of this system is the following: [−1.0,0.0], [0.0,−1.0], [0.0,0.0], [0.0,1.0], [1.0,0.0] and $A={\left[-2.0,2.0\right]}^{2}$;

(31) $$\begin{array}{cc} 0 & =c^{6}+10c^{3}-8={\left(x+iy\right)}^{6}+10{\left(x+iy\right)}^{3}-8\\ \; & =x^{6}-15x^{4}y^{2}+15x^{2}y^{4}-y^{6}+10x^{3}-30xy^{2}-8+\left(6x^{5}y-20x^{3}y^{3}+6xy^{5}+30x^{2}y-10y^{3}\right)i, \end{array}$$

where: $f_{1}\left(x,y\right)=x^{6}-15x^{4}y^{2}+15x^{2}y^{4}-y^{6}+10x^{3}-30xy^{2}-8$, $f_{2}\left(x,y\right)=6x^{5}y-20x^{3}y^{3}+6xy^{5}+30x^{2}y-10y^{3}$ and the set of solutions of this system is the following (approximately): [−2.207,0], [−0.453,−0.785], [−0.453,0.785], [0.906,0], [1.103,−1.911], [1.103,1.911] and A=[−2.3,1.7]×[−2.0,2.0].

**Figure 1:**
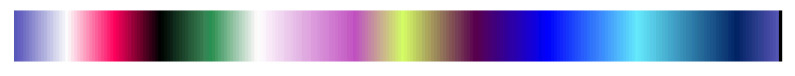
Colour map used in the experiments.

In the experiments, to solve (28) we use the method introduced in Section 2. The same colour map (Figure 1) was used to colour all the obtained polynomiographs. Furthermore, in every experiment we used the following common parameters: m=256, ε=0.01, image resolution 800×800 pixels.

The algorithms used in the experiments were implemented in the C++ programming language. The experiments were conducted on a computer with the Intel Core i7 processor, 8 GB RAM and Linux Ubuntu 18.04 LTS.

### 5.1. The Picard Iteration

**Figure 2:**
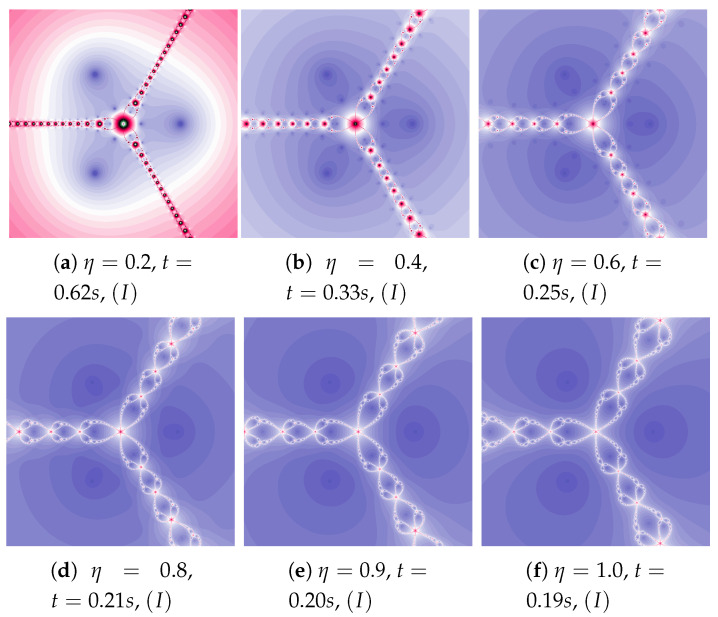
Polynomiographs of the Picard iteration for ω=0.0 and varying η.

The motion of particles depends on the acceleration constant (η) and inertia weight (ω). These parameters control dynamics of a particle behaviour. The polynomiograph visualises particle dynamics and allows analysing parameters impact on the algorithm’s operation. Polynomiographs are created using Algorithm 1. The dynamics visualisations for the Picard iteration using ω=0.0 and varying η are presented in Figure 2. Earlier, in Section 2, we noticed that the proposed method reduces to the relaxed Newton’s method for ω=0 and η≠1 and to the Newton’s method for ω=0 and η=1, so Figure 2a–e present polynomiographs for the relaxed Newton’s method, whereas Figure 2f for the classical Newton’s method. The polynomiograph generation times are also given in Figure 2. The time decreases as the dynamics controlled by the acceleration constant increases.

**Figure 3:**
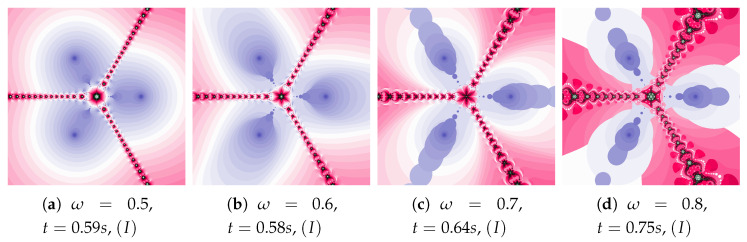
Polynomiographs of the Picard iteration for η=0.1 and varying ω.

Polynomiographs of the Picard iteration for low value of acceleration constant (η=0.1) and varying ω are presented in Figure 3. The increase in the inertia weight results in the increase in particle dynamics. Both too small and too high particle dynamics cause the increase in the polynomiographs creation time. The shortest time was obtained for Figure 3b. Every change of dynamics creates a new image.

**Figure 4:**
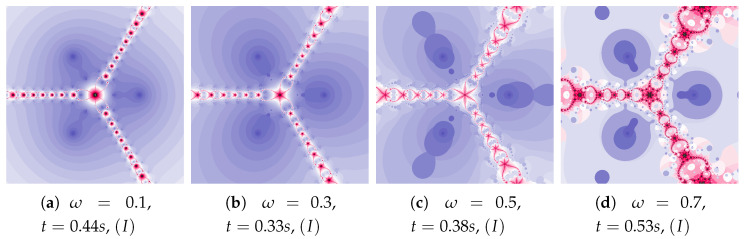
Polynomiographs of the Picard iteration for η=0.3 and varying ω.

The acceleration constant is equal to 0.3 for images in Figure 4. Change in particle’s dynamics is realised by the increase of the inertia weight. As in the previous example, the proper selection of particle’s dynamics minimises the creation time of the polynomiograph (see Figure 4b). The increase in the particle dynamics is expressively illustrated by polynomiographs.

**Figure 5:**
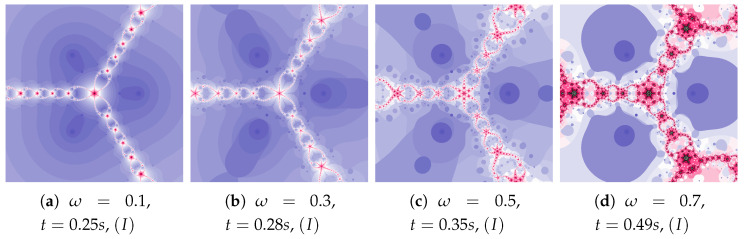
Polynomiographs of the Picard iteration for η=0.5 and varying ω.

In Figure 5 we see polynomiographs generated using the same values of the inertia weight as in Figure 4, but with higher value of the acceleration constant—η=0.5. The increase in the inertia weight causes the increase of the time of image creation. It is the consequence of the excessive growth of particle’s dynamics.

**Figure 6:**
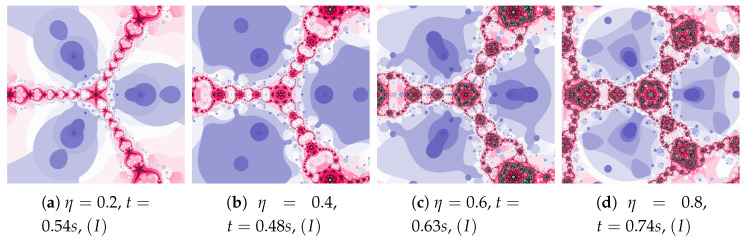
Polynomiographs of the Picard iteration for ω=0.7 and varying η.

Polynomiographs of the Picard iteration for a constant value of the inertia weight (ω=0.7) and varying acceleration constant are presented in Figure 6. For the images in Figure 6c,d the dynamics presented by the polynomiographs is high (the parts of the image are blurred—there are non-contrast patterns created by the points). Moreover, let us notice that the generation time for Figure 6a is lower than the time obtained for Figure 2a (0.62 s—the Picard iteration with ω=0, η=0.2). Thus, by increasing the inertia we were able to obtain a shorter time than in the case of the relaxed Newton’s method with the same value of acceleration.

**Figure 7:**
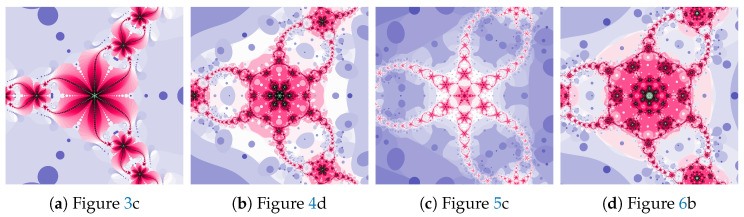
Magnification of the central part of selected polynomiographs of the Picard iteration.

The magnifications of the central part of selected polynomiographs of the Picard iteration are presented in Figure 7. Changes in particle dynamics paint beautiful patterns. They can be an inspiration for creating mosaics.

### 5.2. The Mann Iteration

**Figure 8:**
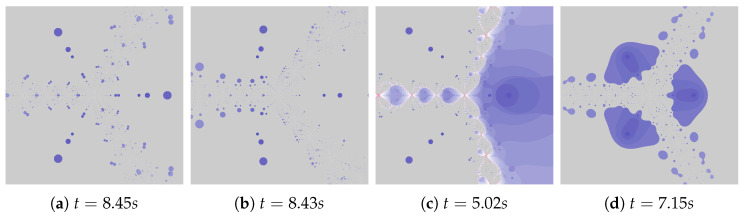
Polynomiographs of the Mann iteration with the fixed reference point for α=0.9, ω=0.3, η=0.5. The positions of the reference point: (**a**) [0,0], (**b**) [−1,0], (**c**) [1,0] (one of the roots), (**d**) the starting point.

Su and Qin in [45] discussed a strong convergence of iterations in the case of a fixed reference point. We start the examples for the Mann iteration with an example showing the behaviour of the Mann iteration implementing a fixed reference point. The results are presented in Figure 8. For polynomiograph presented in Figure 8a the fixed reference point is in its centre [0,0], whereas in Figure 8b this point is in the position [−1,0]—the behaviour of the algorithm is similar in both cases. For the polynomiograph in Figure 8c the fixed reference point is placed in the root position [1,0] (in the best position). It causes a significant improvement in the algorithm operation near the root position. For polynomiograph presented in Figure 8d, a fixed reference point is the starting point—it results in greater efficiency in the algorithm operation near positions of the roots. A better position of the reference point can significantly improve the algorithm operation—however, it is impossible to determine its best position. In the proposed methods, instead of using a fixed reference point we use the locally best position of the reference point. This position is modified when the new position of the reference point is better than the previous one.

The Mann iteration allows to compare the operation of algorithm I with the Algorithm IIIM. The choice of the best position of the particle will cause the change in the dynamics, it can result in a better convergence of the particle—this mechanics is used in many algorithms. Visualisation is a great tool for presenting this mechanics of algorithm operation.

**Figure 9:**
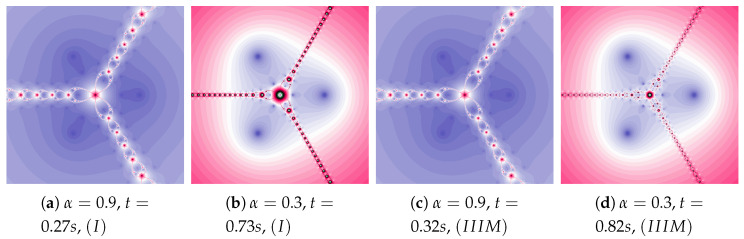
Polynomiographs of the Mann iteration for ω=0.0 and η=0.6 varying α.

Polynomiographs of the Mann iteration for ω=0.0, η=0.6 and varying α are presented in Figure 9. A small value of α causes a reduction in particle’s dynamics. When Algorithm IIIM is used, the areas with the higher dynamics (areas of dynamically changing pattern) are narrowed (compare Figure 9a with Figure 9c).

**Figure 10:**
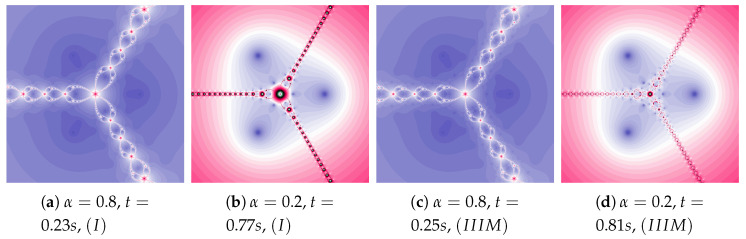
Polynomiographs of the Mann iteration for ω=0.0, η=0.9 and varying α.

The particle dynamics for polynomiographs in Figure 10 is greater than in Figure 9. The high value of the α coefficient limits the effect of selecting the best particle’s position in Algorithm IIIM. Nevertheless, in Figure 10c we observe a narrowing of areas of high dynamics and small changes in the shape of neighbouring areas. Limiting the particle’s dynamics by decreasing in the value of the α coefficient affects the visualisation of the influence of the best particle position on the Algorithm IIIM operation (compare Figure 10b and Figure 10d)—similarly to Figure 9d.

**Figure 11:**
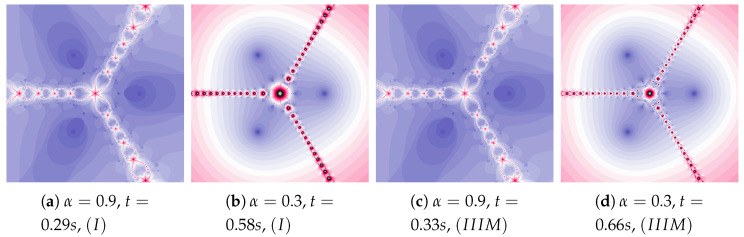
Polynomiographs of the Mann iteration for ω=0.3, η=0.5 and varying α.

In the examples presented in Figure 11, the increase in particle dynamics is obtained by including inertia. The increase in particle dynamics is evident in polynomiographs in Figure 11a,c. As in the previous cases, Algorithm IIIM makes changes visible only in areas with higher dynamics.

**Figure 12:**
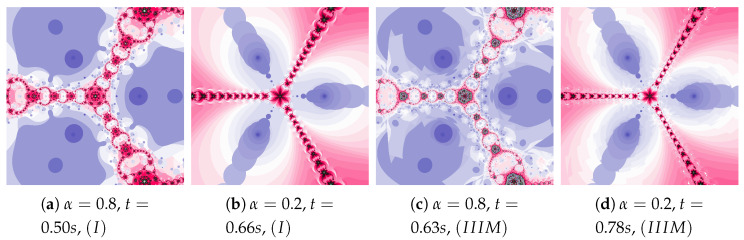
Polynomiographs of the Mann iteration for ω=0.7, η=0.5 and varying α.

Another increase in the inertia weight (ω=0.7) causes the increase in dynamics shown in images in Figure 12a,b. The changes in dynamics caused by the Algorithm IIIM operation are visible in the whole area of polynomiograph (see Figure 12c,d). The time of creation of polynomiograph in Figure 12d, when compared to Figure 12b, is shorter. The proposed modification of the algorithm can accelerate finding the solution.

**Figure 13:**
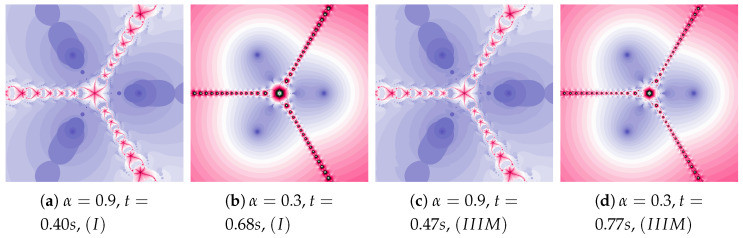
Polynomiographs of the Mann iteration for ω=0.5, η=0.3 and varying α.

In polynomiographs in Figure 13, the particle dynamics are limited by the small value of η (η=0.3). The changes in dynamics caused by the Algorithm IIIM are in areas of high dynamics, the neighbourhood of these areas and places where the particle gets high velocity (the long distance from the centre of the image). This is clearly visible in images in Figure 13c,d.

**Figure 14:**
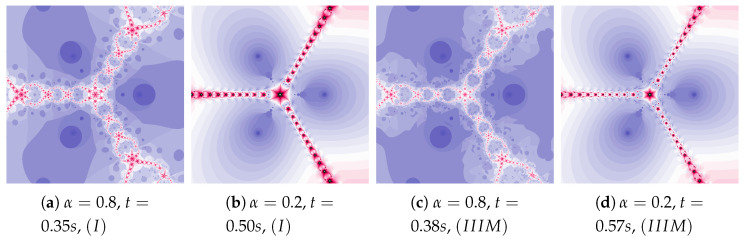
Polynomiographs of the Mann iteration for ω=0.5, η=0.7 and varying α.

Polynomiographs presented in Figure 14 show the increase in dynamics due to the increase in the acceleration constant (η=0.7). The dynamics is high for image in Figure 14a, and in Figure 14b the dynamics is limited by the small value of α. Algorithm IIIM clearly influences the change in particle’s dynamics—Figure 14c. The dynamics caused by the Algorithm IIIM operation are much smaller in Figure 14d because the dynamics are limited by the small value of α.

**Figure 15:**
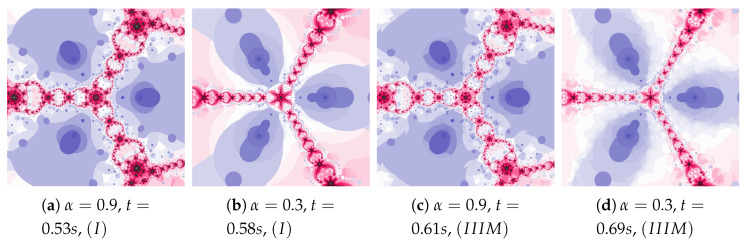
Polynomiographs of the Mann iteration for ω=0.7, η=0.6 and varying α.

The inertia weight has much greater effect on particle dynamics than the acceleration constant. The dynamics presented in Figure 15 is high due to the high value of ω and η. The dynamics changes, caused by the best position of the particle, are clearly visible.

**Figure 16:**
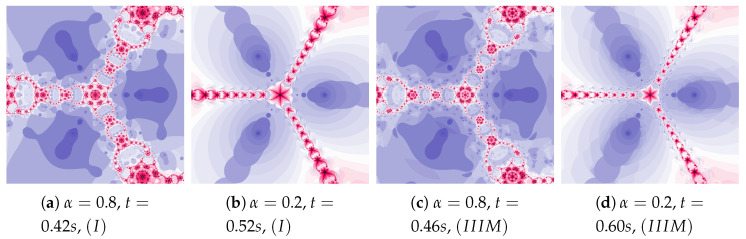
Polynomiographs of the Mann iteration for ω=0.6, η=0.8 and varying α.

Similarly, as in the example presented in Figure 15, the dynamics in Figure 16 are shaped by the high value of ω and η (ω=0.6, η=0.8). Additionally, in this case the dynamics paints some interesting images.

**Figure 17:**
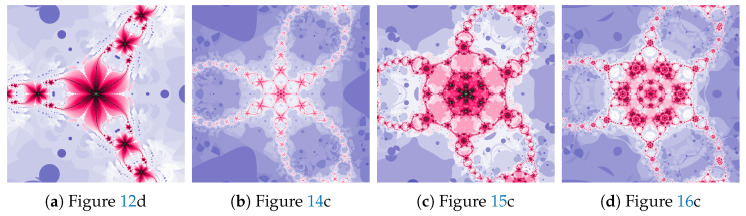
Magnification of the central part of selected polynomiographs of the Mann iteration.

The magnifications of the central part of selected polynomiographs of the Mann iteration and its modifications are presented in Figure 17. Changes in the particle dynamics are visualised by colourful mosaics, which can be used in design to create ornaments.

### 5.3. The Ishikawa and the Das–Debata Iterations

Ishikawa and Das–Debata iterations use a reference point to determine a new particle position. In the presented analysis, base Algorithm *I* and its three modifications are used: Algorithm II using the best position of the reference point, the Algorithm III using the best position of the particle, and both these modifications in the Algorithm IV.

**Figure 18:**
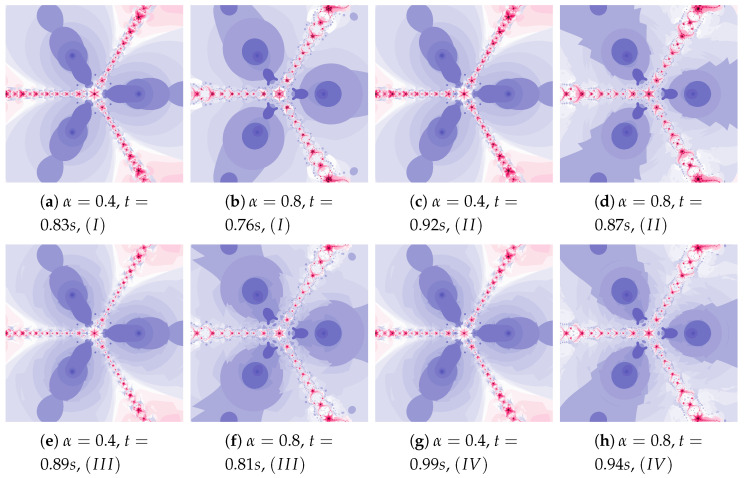
Polynomiographs of the Ishikawa iteration for β=0.4, $\omega_{1}=0.7$, $\eta_{1}=0.3$, $\omega_{2}=0.7$, $\eta_{2}=0.3$ and varying α.

Figure 18 shows polynomiographs of all four algorithms for two values of α (α=0.4 and α=0.8). The low α value limits the influence of the reference point, so images in Figure 18a and Figure 18c are very similar. Algorithm III using the best particle position has a significant influence on the form of the polynomiograph. It results in a strong similarity between images in Figure 18e and Figure 18g. The dynamics shown in Figure 18b is high. The influence of the best reference point is shown in Figure 18d because of the large value of α. Changes in dynamics are also visible in Figure 18f created by the algorithm using the best position of particle. Each of the algorithms has a different influence on the dynamics of the particle. These features are combined in Algorithm IV—it clearly visualises the image in Figure 18h.

**Figure 19:**
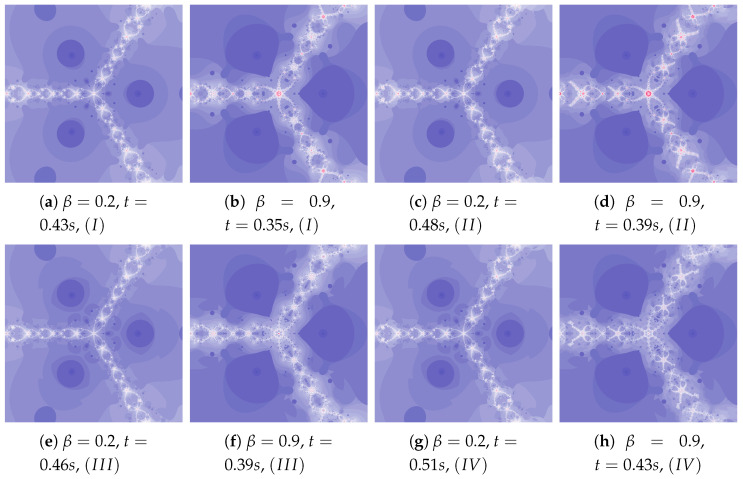
Polynomiographs of the Ishikawa iteration for α=0.7, $\omega_{1}=0.4$, $\eta_{1}=0.8$, $\omega_{2}=0.4$, $\eta_{2}=0.8$ and varying β.

A low value of β reduces the impact of the transformation operator on the dynamics of reference point creation. It has the same effect as the low value of α—for this reason Figure 19a is similar to Figure 19c,e–g. The increase in the β coefficient results in obtaining different features of the images in Figure 19b,d,f,h—the image in Figure 19h combines the features of the images in Figure 19d,f.

The dynamics presented by the polynomiographs depends on the dynamics of the creation of the reference point and its processing. The parameters $\omega_{1}$ and $\eta_{1}$ are responsible for the dynamics of the reference point creation and the parameters $\omega_{2}$ and $\eta_{2}$ are responsible for its processing. The change in these parameters allows to obtain the effects described above.

**Figure 20:**
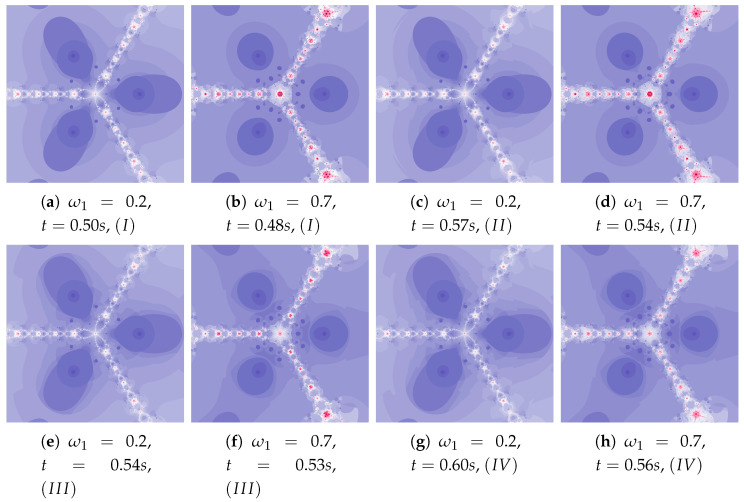
Polynomiographs of the Das–Debata iteration for α=0.6, β=0.7, $\eta_{1}=0.4$, $\omega_{2}=0.5$, $\eta_{2}=0.5$ and varying $\omega_{1}$.

**Figure 21:**
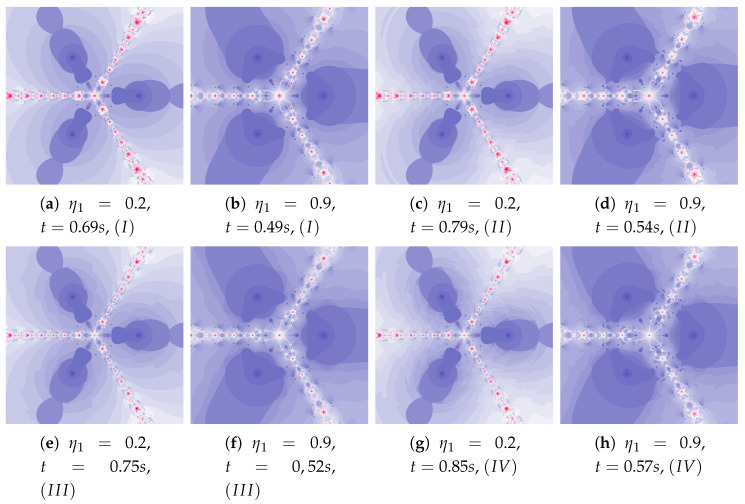
Polynomiographs of the Das–Debata iteration for α=0.5, β=0.6, $\omega_{1}=0.3$, $\omega_{2}=0.6$, $\eta_{2}=0.4$ and varying $\eta_{1}$.

Polynomiographs of the Das–Debata iteration for varying $\omega_{1}$ are presented in Figure 20 and for varying $\eta_{1}$ in Figure 21. Polynomiographs created by Algorithms *I* and II are similar—it means that the influence of choosing the best reference point is small for such selected parameters of the algorithm. The influence of the best position of particle has a significant effect on the form of the polynomiographs created by the Algorithm III—these polynomiographs have many common features with the polynomiographs created by the Algorithm IV (the influence of Algorithm II is small).

**Figure 22:**
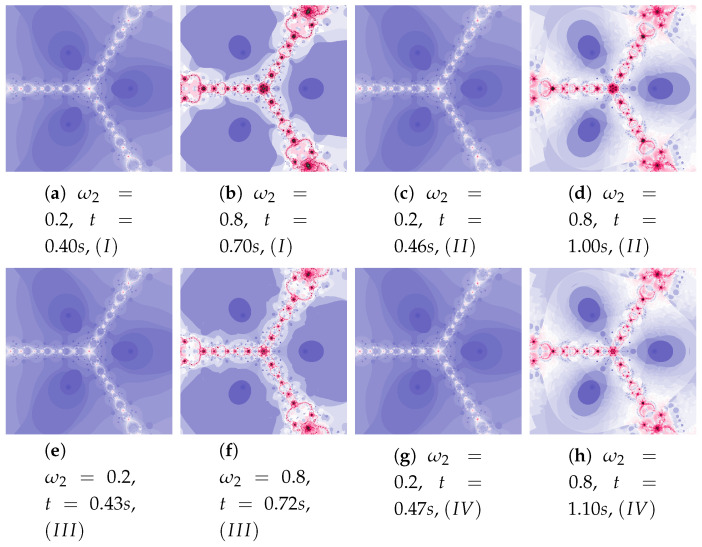
Polynomiographs of the Das–Debata iteration for α=0.9, β=0.7, $\omega_{1}=0.6$, $\eta_{1}=0.2$, $\eta_{2}=0.5$ and varying $\omega_{2}$.

**Figure 23:**
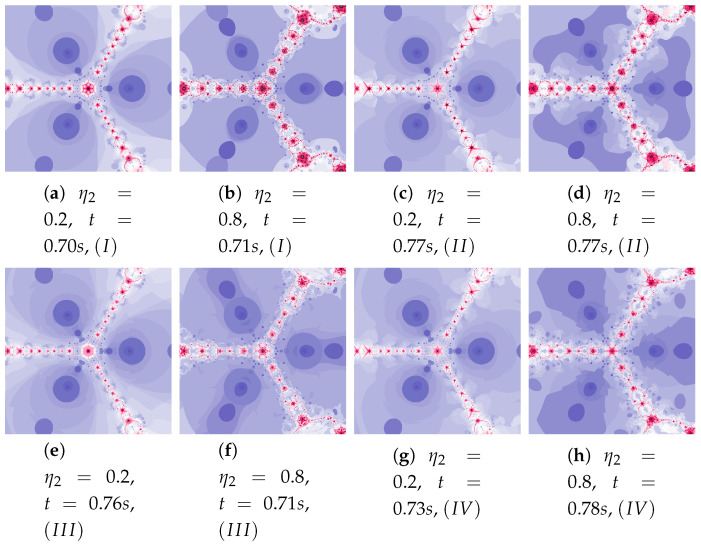
Polynomiographs of the Das–Debata iteration for α=0.9, β=0.7, $\omega_{1}=0.4$, $\eta_{1}=0.2$, $\omega_{2}=0.5$ and varying $\eta_{2}$.

The parameters $\omega_{2}$ and $\eta_{2}$ influence the processing of the reference point. In Figure 22 polynomioographs for varying $\omega_{2}$ and in Figure 23 for varying $\eta_{2}$ are shown. For small values of $\omega_{2}$, polynomiographs presented in Figure 22a,c,e,g are similar—it means, that the operation of all the algorithms is similar. Significant increase in the value of $\omega_{2}$ shows a significant influence of the reference point on the polynomiograph’s creation—Algorithms II and IV similarly create polynomiographs.

All the algorithms make characteristic changes in polynomiographs shown in Figure 23. For high value of $\eta_{2}$ the polynomiographs created by Algorithms II and IV are similar (see Figure 23d).

**Figure 24:**
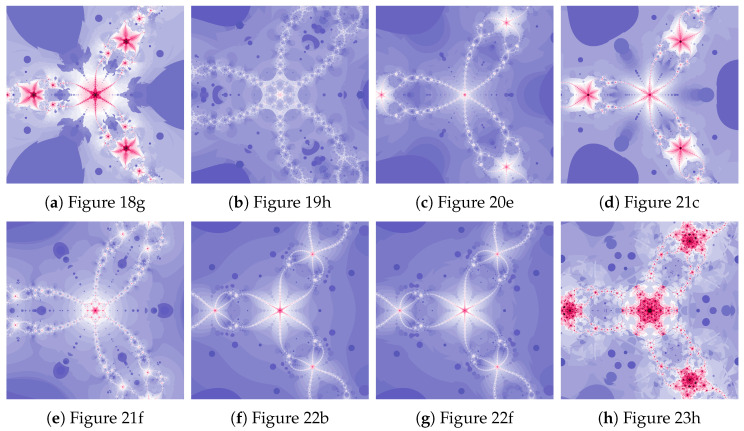
Magnification of the central part of selected polynomiographs of Ishikawa and Das–Debata iterations.

The magnifications of the central part of selected polynomiographs obtained with the Ishikawa and the Das–Debata iterations are presented in Figure 24. Similar to the previous examples these images have artistic features and can be used for instance in ornamentation.

### 5.4. The Agarwal and the Khan–Cho–Abbas Iterations

**Figure 25:**
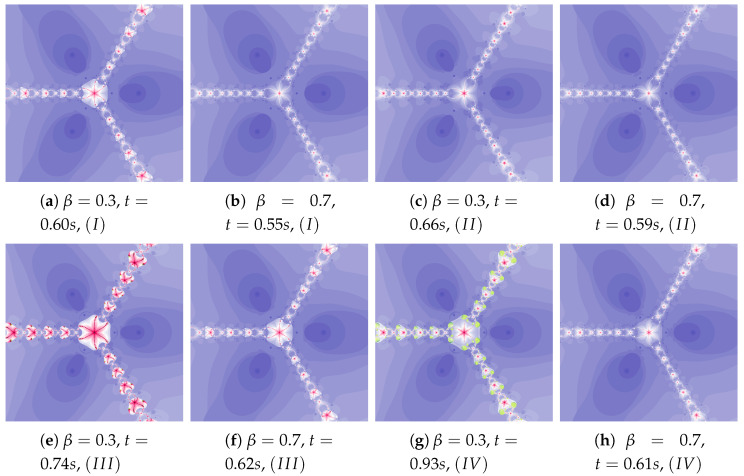
Polynomiographs of the Agarwal iteration for β=0.5, $\omega_{1}=0.4$, $\eta_{1}=0.3$, $\omega_{2}=0.4$, $\eta_{2}=0.3$, $\omega_{3}=0.4$, $\eta_{3}=0.3$ and varying α.

**Figure 26:**
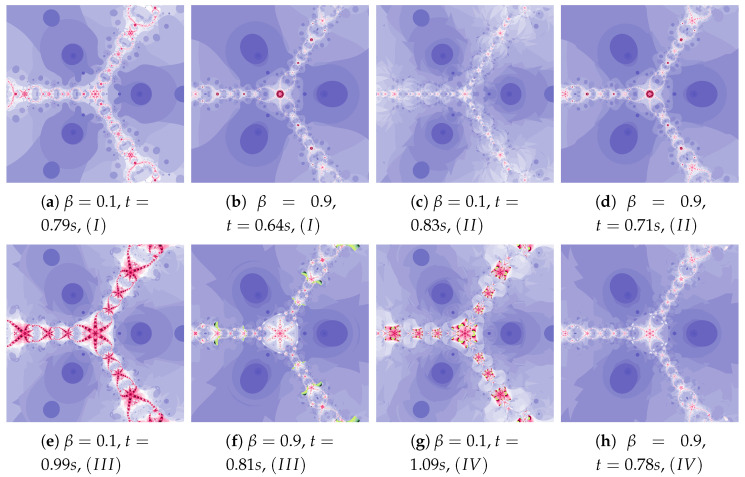
Polynomiographs of the Agarwal iteration for α=0.5, $\omega_{1}=0.5$, $\eta_{1}=0.5$, $\omega_{2}=0.5$, $\eta_{2}=0.5$, $\omega_{3}=0.5$, $\eta_{3}=0.5$ and varying β.

**Figure 27:**
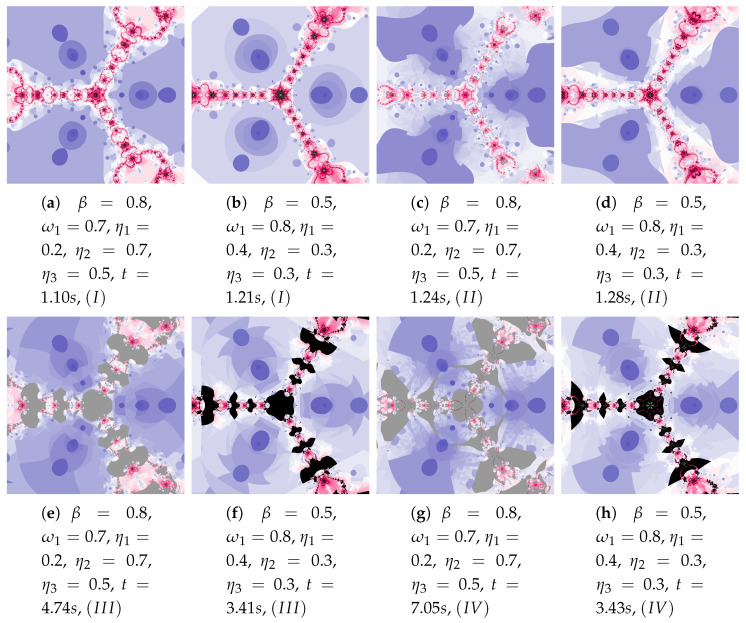
Polynomiographs of the generalised Agarwal iteration for α=0.5, $\omega_{2}=0.6$, $\eta_{2}=0.7$, $\omega_{3}=0.9$.

**Figure 28:**
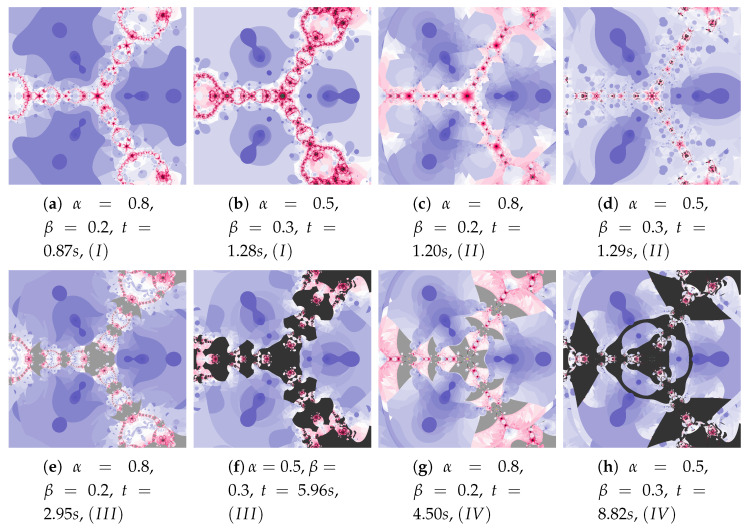
Polynomiographs of the generalised Agarwal iteration for $\omega_{1}=0.3$, $\eta_{1}=0.4$, $\omega_{2}=0.6$, $\eta_{2}=0.7$, $\omega_{3}=0.9$, $\eta_{3}=0.5$.

The generalised Agarwal iteration introduces three operators parameters of which can be independently specified. It allows for a wide range of changes in particle’s dynamics. In addition, α and β parameters allow to determine the impact of these operators on the algorithm work. In Figure 25 polynomiographs of the Agarwal iteration for varying α and in Figure 26 for varying β are presented. Incorrect selection of these parameters can cause that the algorithm does not reach a solution in a given number of iterations—it results in extension of the algorithm’s operation time (see Figure 25e). Nevertheless, the increased particle dynamics gives the opportunity to obtain interesting patterns. Poorly selected particle dynamics can cause a significant increase in the creation time of a polynomiograph. This case is visible in the polynomiographs in Figure 27 and Figure 28 for Algorithms III and IV.

**Figure 29:**
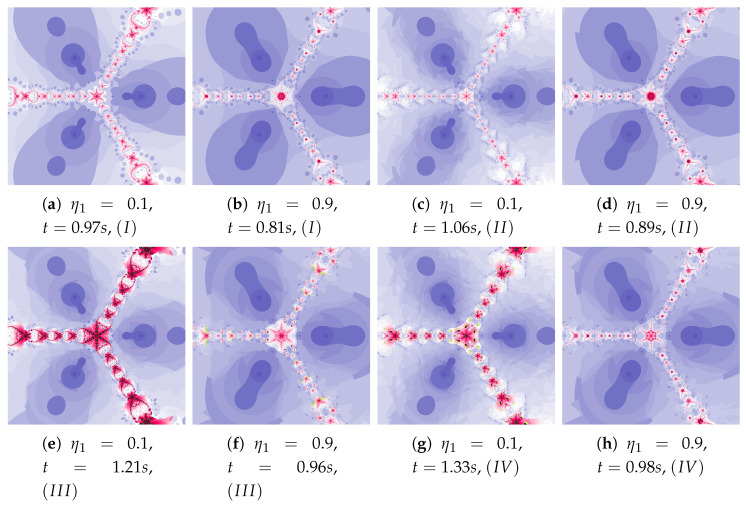
Polynomiographs of the generalised Agarwal iteration for α=0.5, β=0.5, $\omega_{1}=0.6$, $\omega_{2}=0.6$, $\eta_{2}=0.3$, $\omega_{3}=0.6$, $\eta_{3}=0.3$ ($T_{2}=T_{3}$) and varying $\eta_{1}$.

**Figure 30:**
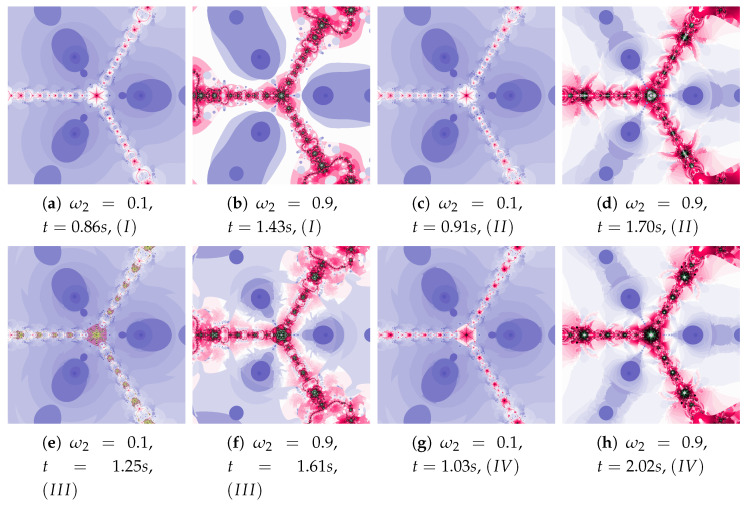
Polynomiographs of the Khan–Cho–Abbas iteration for α=0.5, β=0.5, $\omega_{1}=0.7$, $\eta_{1}=0.2$, $\eta_{2}=0.5$, $\omega_{3}=0.7$, $\eta_{3}=0.2$ and varying $\omega_{2}$.

**Figure 31:**
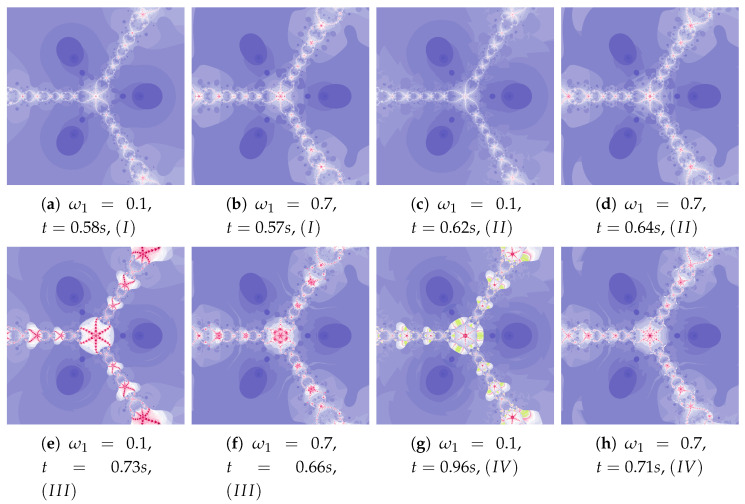
Polynomiographs of the generalised Agarwal iteration for α=0.5, β=0.5, $\eta_{1}=0.6$, $\omega_{2}=0.4$, $\eta_{2}=0.6$, $\omega_{3}=0.4$, $\eta_{3}=0.6$ ($T_{2}=T_{3}$) and varying $\omega_{1}$.

**Figure 32:**
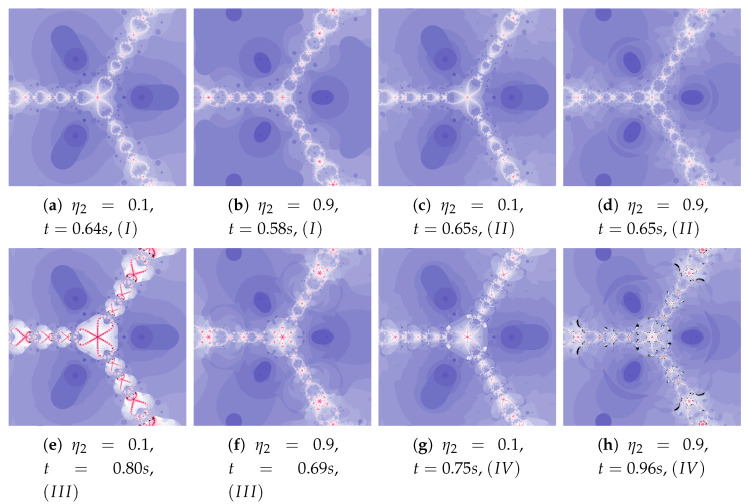
Polynomiographs of the Khan–Cho–Abbas iteration for α=0.5, β=0.5, $\omega_{1}=0.3$, $\eta_{1}=0.7$, $\omega_{2}=0.6$, $\omega_{3}=0.3$, $\eta_{3}=0.7$ and varying $\eta_{2}$.

**Figure 33:**
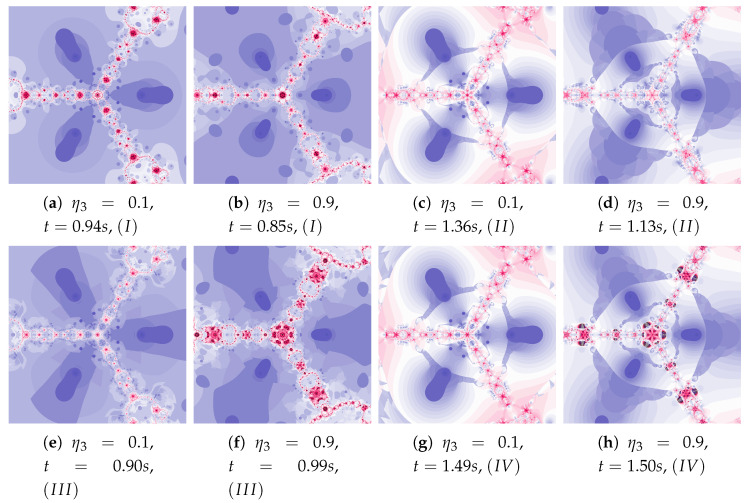
Polynomiographs of the generalised Agarwal iteration for α=0.8, β=0.4, $\omega_{1}=0.2$, $\eta_{1}=0.4$, $\omega_{2}=0.7$, $\eta_{2}=0.7$, $\omega_{3}=0.5$ and varying $\eta_{3}$.

**Figure 34:**
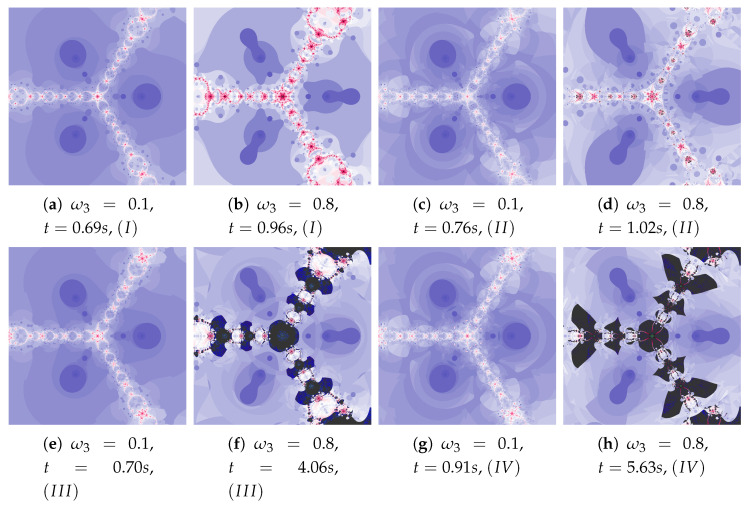
Polynomiographs of the generalised Agarwal iteration for α=0.5, β=0.3, $\omega_{1}=0.4$, $\eta_{1}=0.6$, $\omega_{2}=0.6$, $\eta_{2}=0.7$, $\eta_{3}=0.5$ and varying $\omega_{3}$.

Depending on the choice of parameters, cases that have already been discussed in the previous iterations can be observed. The big similarity of polynomiographs created by Algorithms *I* and II as well as III and IV is observed in Figure 29 and Figure 30 ($\omega_{2}=0.1$). Polynomiographs are similar for all algorithms with the low particle dynamics—this is observed in Figure 31. The strong similarity of polynomiographs is shown in Figure 32 and Figure 33 for Algorithms II and IV—it indicates a strong influence of the reference point on the polynomiographs creation. Next, the strong similarity of polynomiographs is observed in Figure 34 for Algorithm *I* and III—it results from the strong influence of the best particle position on the polynomiographs creation.

**Figure 35:**
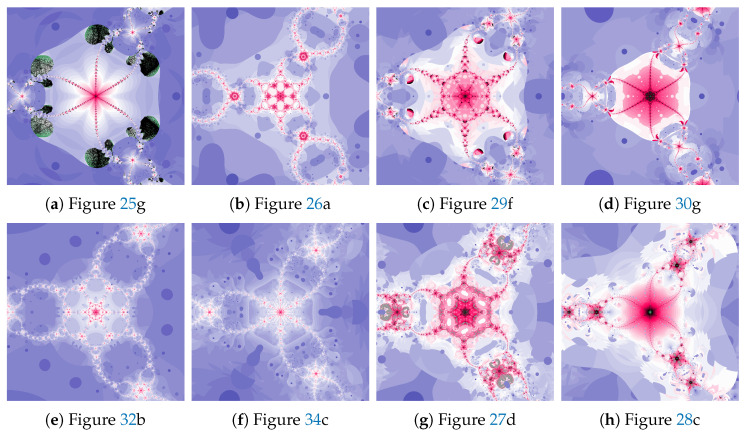
Magnification of the central part of selected polynomiographs of Agarwal and Khan–Cho–Abbas iterations.

The magnifications of the central part of selected polynomiographs of Agarwal and Khan–Cho–Abbas iterations are presented in Figure 35. High dynamics can create interesting patterns, especially for the Agarwal iteration. These patterns can be an artistic inspiration.

### 5.5. Algorithms Operation in the Selected Test Environments

**Figure 36:**
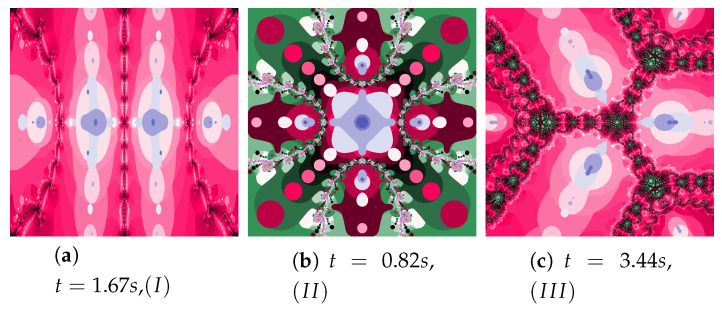
Polynomiographs of the Picard iteration for the polynomiograph settings from the Figure 4d.

**Figure 37:**
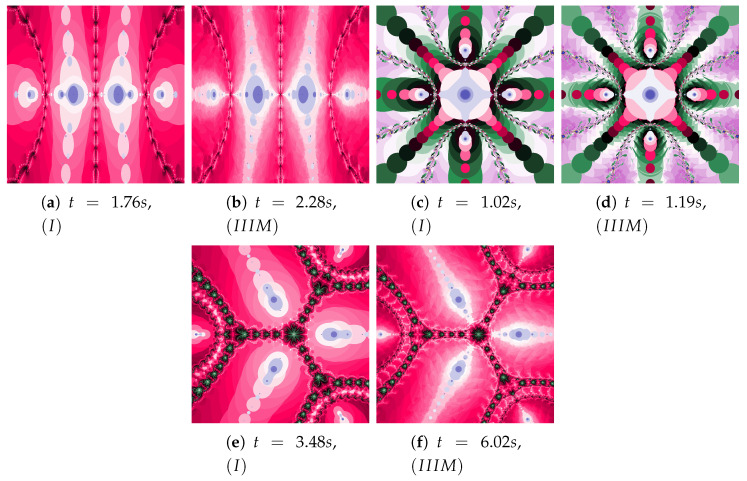
Polynomiographs of the Mann iteration for the polynomiograph settings from the Figure 15b.

**Figure 38:**
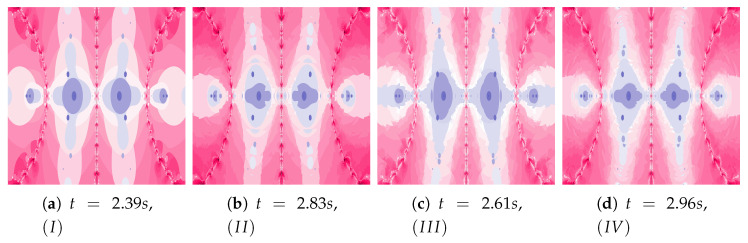

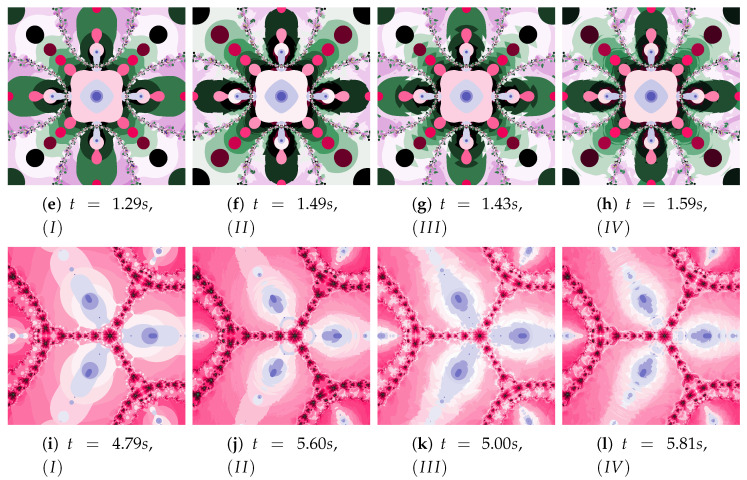
Polynomiographs of the Ishikawa iteration for the polynomiograph settings from the Figure 18b.

**Figure 39:**
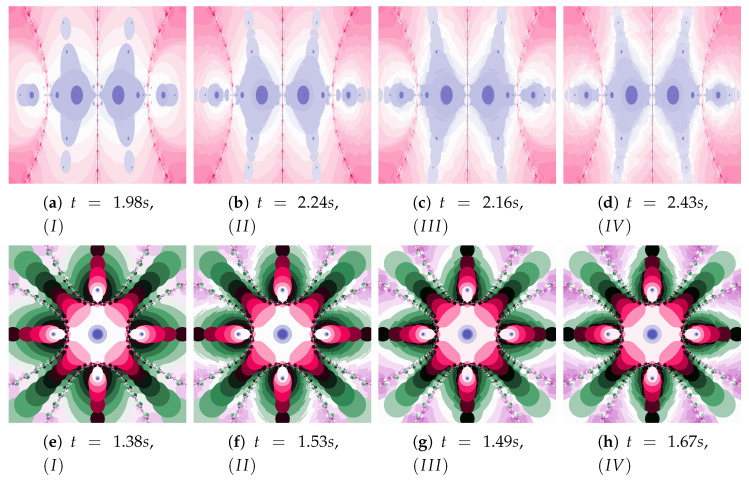

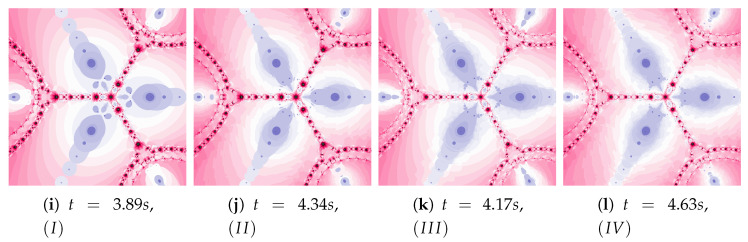
Polynomiographs of the Das–Debata iteration for the polynomiograph settings from the Figure 21a.

**Figure 40:**
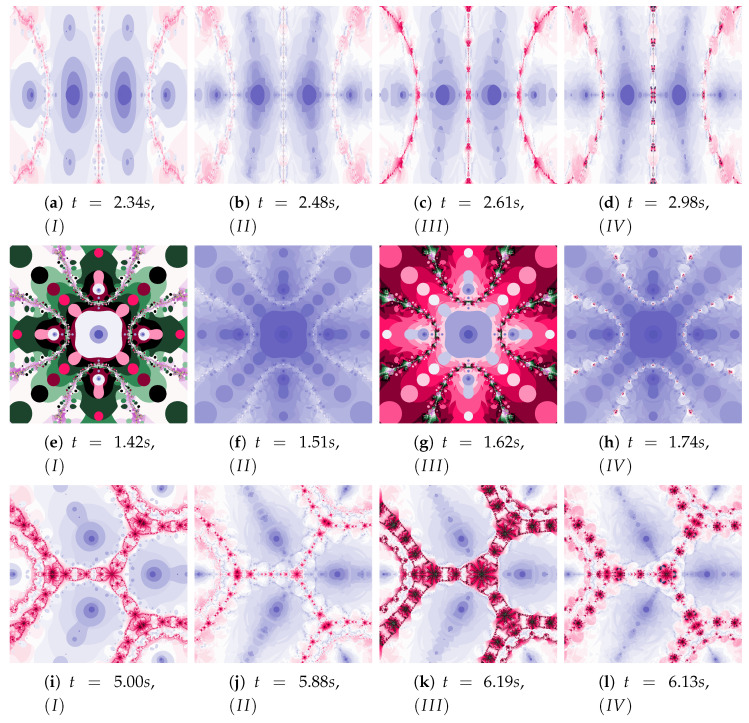
Polynomiographs of the Agarwal iteration for the polynomiograph settings from the Figure 26a.

**Figure 41:**
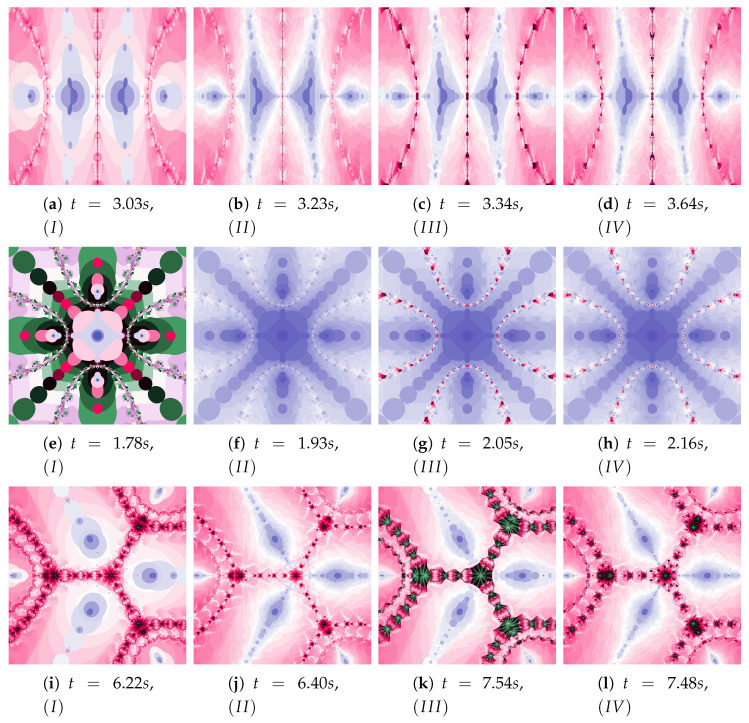
Polynomiographs of the generalised Agarwal iteration for the polynomiograph settings from the Figure 29a.

**Figure 42:**
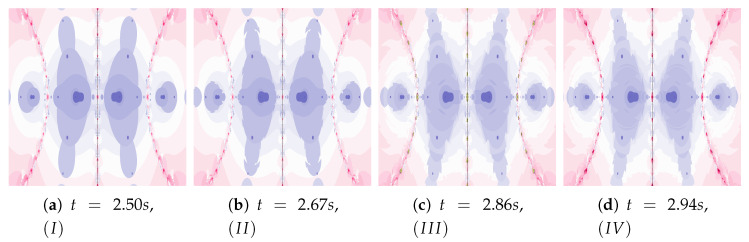

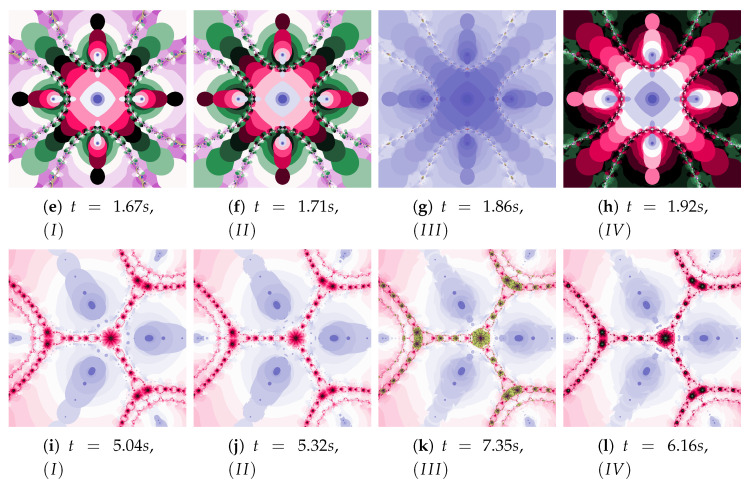
Polynomiographs of the Khan–Cho–Abbas iteration for the polynomiograph settings from the Figure 30a.

The discussion conducted in the previous sections presented in detail the impact of individual factors on the operation of the proposed algorithm. The authors want to supplement this discussion with a presentation of algorithms’ operation for the selected test environments, which we can be found in the world literature and were presented at the beginning of Section 5. Figure 36, Figure 37, Figure 38, Figure 39, Figure 40, Figure 41, Figure 42 present polynomiographs for three test environments and the iterations discussed in the article. They will allow to generalise the observations discussed in the previous sections. In Figure 36 the Picard’s iteration, in Figure 37a,c,e the Mann’s iteration and in sub-figures (a), (e), (i) of Figure 38, Figure 39, Figure 40, Figure 41, Figure 42 polynomiographs realised by the base algorithm are presented. These polynomiographs are characterised by smooth boundaries of areas with different particle dynamics. The implementation of the algorithm’s modification consisting in the selection of position of the best reference point and particle causes the blur and rag of smooth edges of the areas presented on the polynomiographs obtained for the base algorithm. The selection of the best position of the reference point implemented in the Algorithm (II) results in a significant reduction of small elements (details) of the patterns visible on the polynomiograph. It is a significant limitation of particle dynamics, which can be seen in the (b) sub-figures of Figure 38, Figure 39, Figure 40, Figure 41, Figure 42. Figure 37b,d,f and the (c) sub-figures of Figure 38, Figure 39, Figure 40, Figure 41, Figure 42 present polynomiographs of the Algorithm (IIIM and III) selecting the best particle position. The operation of the algorithm results in much better presentation of small details in the polynomiograph—these are the areas in which the particle wants to achieve a worse position. Polynomiographs obtained by using the algorithm denoted by (IV), i.e., with the selection of the best reference point and the best particle position, are shown in the (d) sub-figures of Figure 38, Figure 39, Figure 40, Figure 41, Figure 42. We can observe both features of the polynomiographs from the sub-figures (b) and (c) of Figure 38, Figure 39, Figure 40, Figure 41, Figure 42. However, the polynomiograph’s features have a dominant influence for choosing the best particle position. The analysis of the changes in particle dynamics resulting from the applied algorithm can also be performed by analysing the simulation time. The best position of the reference point has a significant impact on the reduction of particle dynamics. One can only complete the observations regarding the test environments. In environments $p\left(c\right)=c^{3}-1$, $p\left(c\right)=c^{4}-10c^{2}+9$, $p\left(c\right)=c^{6}+10c^{3}-8$ the particle obtains similar ranges of dynamics, whereas for the $p\left(c\right)=c^{5}-c$ environment the particle achieves larger dynamics ranges.

## 6. Conclusions

The paper proposes a modification of the Newton’s method and introduces algorithms based on it. Similar approaches are presented in many optimisation algorithms which require parameters tuning, e.g., the PSO algorithm. The discussion presented in the article allows showing inertia weight and the acceleration constant impact on the operation of the proposed algorithms. Both too large and too small dynamics of particle can have an adverse influence on the algorithm’s operation. Polynomiography is a tool illustrating the behaviour of particles. It also creates an artistic mosaics and allows obtaining images that can be named as art.

## Figures and Tables

**Table 1:** Computational order of convergence for system given by $f_{1}\left(x,y\right)=x^{3}-3xy^{2}-1$, $f_{2}\left(x,y\right)=3x^{2}y-y^{3}$ solved by (8).

Parameters (ω,η)	$z_{0}=\left[-2.5,2.5\right]$	$z_{0}=\left[-2,-2\right]$	$z_{0}=\left[2.5,2.5\right]$
(0.0,1.0)	2.06	2.01	2.03
(0.1,0.9)	1.15	1.93	0.91
(0.1,0.8)	1.10	0.61	0.31
(0.1,0.7)	0.95	1.18	2.43
(0.1,0.6)	1.28	1.30	1.34
(0.2,0.7)	1.67	2.47	2.79
(0.2,0.6)	0.79	1.96	1.18
(0.25,0.6)	1.52	2.01	0.94
(0.3,0.6)	0.54	1.68	2.82
